# Atypical brain lateralisation in the auditory cortex and language performance in 3- to 7-year-old children with high-functioning autism spectrum disorder: a child-customised magnetoencephalography (MEG) study

**DOI:** 10.1186/2040-2392-4-38

**Published:** 2013-10-08

**Authors:** Yuko Yoshimura, Mitsuru Kikuchi, Kiyomi Shitamichi, Sanae Ueno, Toshio Munesue, Yasuki Ono, Tsunehisa Tsubokawa, Yasuhiro Haruta, Manabu Oi, Yo Niida, Gerard B Remijn, Tsutomu Takahashi, Michio Suzuki, Haruhiro Higashida, Yoshio Minabe

**Affiliations:** 1 Research Centre for Child Mental Development, Kanazawa University, Kanazawa, Japan; 2 Department of Psychiatry and Neurobiology, Graduate School of Medical Science, Kanazawa University, Kanazawa, Japan; 3 Higher Brain Functions and Autism Research, Department of Child Development, United Graduate School of Child Development, Osaka University, Kanazawa University and Hamamatsu University School of Medicine, Osaka University, Osaka, Japan; 4 International Education Centre, Kyushu University, Fukuoka, Japan; 5 Department of Anaesthesiology, Graduate School of Medical Science, Kanazawa University, Kanazawa, Japan; 6 Department of MEG, Yokogawa Electric Corporation, Tokyo, Japan; 7 Department of Neuropsychiatry, University of Toyama, Toyama, Japan

**Keywords:** Autism spectrum disorder (ASD), Laterality index, Magnetoencephalography (MEG), P50m, Young children

## Abstract

**Background:**

Magnetoencephalography (MEG) is used to measure the auditory evoked magnetic field (AEF), which reflects language-related performance. In young children, however, the simultaneous quantification of the bilateral auditory-evoked response during binaural hearing is difficult using conventional adult-sized MEG systems. Recently, a child-customised MEG device has facilitated the acquisition of bi-hemispheric recordings, even in young children. Using the child-customised MEG device, we previously reported that language-related performance was reflected in the strength of the early component (P50m) of the auditory evoked magnetic field (AEF) in typically developing (TD) young children (2 to 5 years old) [*Eur J Neurosci* 2012, **35**:644–650]. The aim of this study was to investigate how this neurophysiological index in each hemisphere is correlated with language performance in autism spectrum disorder (ASD) and TD children.

**Methods:**

We used magnetoencephalography (MEG) to measure the auditory evoked magnetic field (AEF), which reflects language-related performance. We investigated the P50m that is evoked by voice stimuli (/ne/) bilaterally in 33 young children (3 to 7 years old) with ASD and in 30 young children who were typically developing (TD). The children were matched according to their age (in months) and gender. Most of the children with ASD were high-functioning subjects.

**Results:**

The results showed that the children with ASD exhibited significantly less leftward lateralisation in their P50m intensity compared with the TD children. Furthermore, the results of a multiple regression analysis indicated that a shorter P50m latency in both hemispheres was specifically correlated with higher language-related performance in the TD children, whereas this latency was not correlated with non-verbal cognitive performance or chronological age. The children with ASD did not show any correlation between P50m latency and language-related performance; instead, increasing chronological age was a significant predictor of shorter P50m latency in the right hemisphere.

**Conclusions:**

Using a child-customised MEG device, we studied the P50m component that was evoked through binaural human voice stimuli in young ASD and TD children to examine differences in auditory cortex function that are associated with language development. Our results suggest that there is atypical brain function in the auditory cortex in young children with ASD, regardless of language development.

## Background

Currently, the prevalence of autism spectrum disorder (ASD) is estimated to be greater than 1% [1,2]. ASD is an umbrella term for a wide variety of disorders that share common symptoms and aetiologies (i.e., autism, pervasive developmental disorder and Asperger disorder) [3]. There is a wide variety of symptoms, behaviours and types of disorders, as well as considerable individual variation. Although language impairment is not a core feature of ASD, the failure to develop sophisticated language is one of the earlier signs of this disorder [4-6].

Brain leftward lateralisation has long been one of the intriguing properties of human brain development that is associated with language acquisition [7-14]. Compared with TD children, participants with ASD have been reported to possess atypical brain lateralisation as detected by various neuroimaging methods [15-20]. From the perspective of cortical responses to speech sounds in young children or toddlers with ASD, two recent fMRI studies suggested aberrant right lateralisation by demonstrating a trend toward greater recruitment of regions of the right hemisphere during speech stimulation [18,20]. In addition, our recent study using child-sized MEG demonstrated a rightward-lateralised neurophysiological network in conscious young children (including children aged 3–4 years) with ASD while viewing a video programme with speech narration [21]. From an anatomical perspective, the rightward asymmetry of the cortical volume in the auditory association area (i.e., posterior superior temporal gyrus and planum temporale) was also shown in children with ASD [16,22]. These recent studies suggested that aberrant rightward brain function for human speech is one of the physiological hallmarks of ASD that is present at very young ages.

In studies that use magnetoencephalography (MEG), the mid-latency auditory evoked field (AEF) comprises the P50m, N100m and P200m components. The P50m (P1m) is one of the mid-latency components and corresponds to the P50 (P1) in electroencephalography (EEG) studies. While the P50(m) has been widely used to examine sensory gating [23], the P50(m) is a prominent component with shorter inter-stimulus intervals (ISIs) in young children [24-27], and it can be used to provide insight into the development of auditory processing in real-world environments (i.e., auditory information at rapid rates). Although the P50m component was thought to reflect a lower level auditory processing, such as a sensory gating system [28], recent studies have demonstrated that language impairment [29] and the intervention effect [30] were also reflected in the strength of the AEFs (P50m) in young children (5 to 7 years old). In these studies, Pihko and colleagues reported that 6- to 7-year-old children who have language impairments showed weaker P50m responses evoked by syllable stimuli (i.e., /da/ /ba/) compared with children who have normal language development [29]. Furthermore, they investigated the plastic changes in the AEF components in 6- to 7-year-old children after a phonological intervention programme, which comprises the following: (1) speech and articulation exercises, (2) phoneme discrimination exercises and (3) exercises that train phonological and linguistic awareness and rapid processing. Intriguingly, a significant increment in the P50m response was observed after the phonological intervention programme [30]. Furthermore, in our recent study [31], we investigated the relationships between the P50m component and several language-related subtests in the Kaufman Assessment Battery for Children (K-ABC) [32] (i.e., “number recall”, “expressive vocabulary” and “riddles”). “Number recall” reflects the ability for language phonological repetition, “expressive vocabulary” reflects the expressive ability to speak the correct names of objects and illustrations and “riddles” reflects ability at language conceptual inference [32]. In our recent study, a significant correlation was found between the P50m component and performance on the “riddles”, whereas no significant correlation was found with the other K-ABC language subtests. Even though the P50m component was thought to reflect a lower-level sensory processing step such as auditory input change detection for speech-like signals in adult humans [33,34], our recent study demonstrated that the P50m components were correlated with a higher-order task reflecting inferential language processing (i.e., “riddle”) in preschool children [31]. These results may be explained by age-related changes in myelination, synaptic refinement and cortical fibre density [35-38], which underlie the age-related changes in amplitude and latency of P50m components. Additionally, myelination of the auditory system is followed by myelination in language-related brain areas in young children [39], which enhances a long-range brain network and contributes to higher-level language performance in young children. Therefore, P50m evoked by voice stimuli appeared to reflect brain maturation in association with a higher-order language development (e.g., performance of the K-ABC subtest “riddles”) in young children.

According to our previous study, the N100m component is a less detectable component that is found in MEG-equivalent current dipole (ECD) methods in 2- to 5-year-old children [31]. By contrast, the P50m is a prominent component, especially during childhood [24-27], whereas N100m and P200m become more prominent after 9–10 years of age [24]. Therefore, in the present study, we focused on the P50m by using MEG ECD methods, which can yield the absolute values of the brain response in the auditory cortex.

As far as we know, no previous MEG studies have focused on this prominent component (i.e., P50m) simultaneously in both hemispheres in younger children with ASD (age 6 and under), whereas a number of auditory electrophysiological studies have primarily focused on assessing atypical auditory processing in other middle-time components [e.g., N100m or mismatch field (MMF)] in older children with ASD (older than 6 years) [40-45].

A bilateral assessment of young children using simultaneous dipole source analysis revealed that AEF is severely limited for small head sizes, which has potentially restricted previous attempts at P50m studies of individuals at this age. The smaller head size of the young children certainly presents longer distances between the MEG sensors and the bilateral auditory cortices, which severely diminishes the signal-to-noise ratio because the magnetic field strength diminishes with increasing distances from the source. To overcome this problem, in the present study, we used a child-customised MEG device (PQ 1151R; Yokogawa/KIT, Kanazawa, Japan) in which the MEG sensors were positioned as close to the head as possible for optimal recording in young children, as previously reported [7,31]. Using this novel device, we investigated the early P50m component that was evoked through binaural human voice sounds in young ASD and TD children to examine differences in brain function in the auditory cortex of children with ASD compared to TD children. The responses that were examined are associated with language development.

To investigate childhood auditory function in real-world environments, we employed speech stimuli with a short ISI, despite the considerable refractoriness that this causes (i.e., auditory information at rapid rates), and we focussed on the values of the P50m component in the right and left hemispheres, respectively. Given that a shortened refractory period is reflected in higher P50m amplitude and shorter P50m latency, these physiological indices must represent brain maturation in developing children. We investigated how this neurophysiological index in each hemisphere is correlated with language development in young children with ASD and TD children.

## Methods

### Participants

The clinical group included 35 children with ASD (6 girls and 29 boys) at 40–93 months of age, who were recruited from Kanazawa University and prefectural hospitals in the Kanazawa or Toyama area. The ASD diagnosis was made by a psychiatrist and a clinical speech therapist. The speech therapist, who is well trained and has the Autism Diagnostic Observational Schedule research license as well as more than 5 years of experience in ASD treatment, employed the Autism Diagnostic Observational Schedule, Generic (ADOS-G) [46]. The definitive diagnosis of ASD was made by the psychiatrist, who has more than 10 years of experience in ASD, using the Diagnostic Interview for Social and Communication Disorders (DISCO) [47] and the DSM-IV criteria (the American Psychiatric Association, 1994) at the time of the MEG and the Kaufman Assessment Battery for Children (K-ABC) data acquisition. All the children with ASD included in this study satisfied the diagnosis of childhood autism (*n* = 25), atypical autism (*n* = 4) or Asperger’s syndrome (*n* = 6) using the DISCO. Children who were below the ADOS cutoff levels were included in the present study if they met the criteria for ASD using both the DSM-IV criteria and DISCO (i.e., 6 children). A total of 35 TD children (6 girls and 29 boys) ranging from 37 to 85 months old, with no reported behavioural or language problems, were used as controls. The TD children were matched to the ASD subjects according to age in months. All the TD subjects were native Japanese children with no previous or existing developmental, learning or behavioural problems according to information obtained from a questionnaire completed by their parents. All the participants had normal hearing abilities according to available medical records. Left- or right-hand dominance was determined based on their preference when handling objects, and the following results were obtained: TD children (right = 35, left = 0, both = 0) and children with ASD (right = 27, left = 2, both = 6). There were no significant differences in head size between the two groups.

All of the children participated separately in cognitive tasks and MEG measurements over a 2-day period. On the first day, the participants performed cognitive tests and were introduced to the environment for the MEG measurement. The actual MEG measurements were performed on the second day. The parents agreed to the participation of their child in the study with full knowledge of the experimental nature of the research. Written informed consent was obtained prior to participation in the study. The Ethics Committee of Kanazawa University Hospital approved the methods and procedures, all of which were performed in accordance with the Declaration of Helsinki.

### Cognitive and language performance measurements

The children were subjected to the Japanese adaptation of the Kaufman Assessment Battery for Children (K-ABC), which is typically used to assess the cognitive skills of children aged 30–155 months. To confirm the standardised score of the mental processing and achievement scales in children, subtests that were complementary to the age (in months) of the children were used in this battery. Because we have demonstrated the significant correlation between the early component of the AEF (i.e., the P50m intensity) and the performance of a language-related task (i.e., a subtest of K-ABC ‘riddles’) in our previous study [31], we employed this subtest ‘riddles’ in this study. In the riddle task, children were required to answer the examiner’s question, such as “Which fruit has a rounded shape with a depression at the top where the stem is attached? The colour of the skin can be either red, green, yellow, or a combination of these colours”. In this case, the answer is “an apple”. The riddle task consists of 32 questions, which are presented in ascending order of difficulty. The linguistic level is defined by the child’s degree of achievement. The K-ABC ‘riddles’ subtest reflects conceptual language inference abilities [32], and our recent study demonstrated that ‘riddles’ was the weakest subtest for the children with ASD compared with the TD children [48]. There were no significant differences in the K-ABC mental processing scale/achievement scale between groups (Table 1).

**Table 1:** Demographic characteristics of the participants whose P50m dipole sources were reliably estimated in both hemispheres

	**TD**	**ASD**	***t***	***p***
Number of subjects	33	30		
Gender (male/female)	27/6	25/5		
Chronological age (months)	67.4 (10.7)	66.9 (12.0)	0.15	n.s.
Head size (cm)	51.5 (1.5)	51.0 (1.8)	1.23	n.s.
Handedness (right)	33	24		
K-ABC				
Mental Processing Scale	97.4 (12.8)	94.3 (18.9)	0.75	n.s.
Achievement Scale	96.2 (12.2)	95.3 (21.8)	0.21	n.s.
ADOS				
Communication	—	3.9 (2.1)		
Reciprocal Social Interaction	—	7.2 (2.3)		

K-ABC, Kaufman Assessment Battery for Children. ADOS, Autism Diagnostic Observation Schedule.

The values are the mean (standard deviation) for chronological age, head size, scales on the K-ABC and scores of ADOS.

n.s., no significance.

### Magnetoencephalography recordings

**Figure 1:**
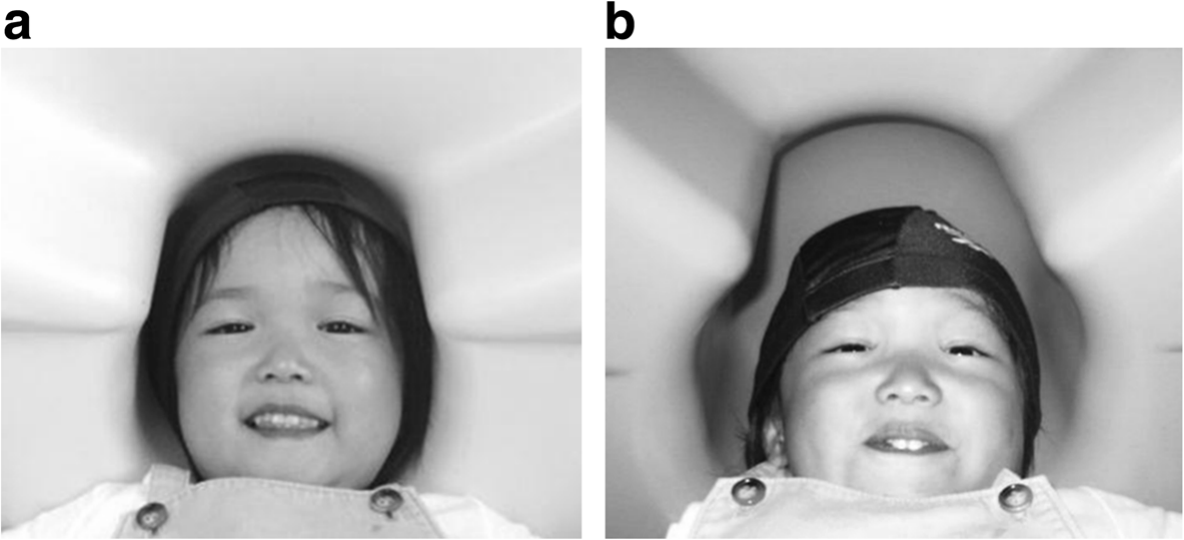
**Comparison between child-sized MEG and adult-sized MEG for head size of young children. (a)** A customised, child-sized MEG. **(b)** A conventional, adult-sized MEG.

The conditions used were similar to those detailed in our previous study [31]. The MEG data were recorded using a 151-channel SQUID (Superconducting Quantum Interference Device) whole-head coaxial gradiometer MEG system for children (PQ 1151R; Yokogawa/KIT, Kanazawa, Japan; Figure 1) in a magnetically shielded room (Daido Steel, Nagoya, Japan), which was installed at the MEG Centre of Yokogawa Electric Corp. (Kanazawa, Japan). The custom child-sized MEG system facilitates the measurement of brain responses in young children, which would be difficult to obtain using conventional adult-sized MEG systems. The child MEG system ensures that sensors are easily and effectively positioned within range of the brain and that head movement is constrained [49]. We determined the position of the head within the helmet by measuring the magnetic fields after passing currents through coils attached at three locations on the head surface, which were used as fiduciary points with respect to the landmarks (bilateral mastoid processes and nasion). Although we could not determine whether the individual head shape would influence the accuracy of the dipole estimation, for calculating ECD without magnetic resonance imaging anatomical data, a spherical model of the volume conductor was fitted to the head of each subject and was confirmed to be located in the centre of the MEG helmet through measurements at three locations on the head surface, as described in our previous study [31]. An examiner remained in the room to encourage the children and to prevent movement throughout the analysis. Stimuli were presented while the children lay in the supine position on the bed and viewed silent video programmes projected onto a screen.

### Auditory evoked field stimuli and procedures

**Figure 2:**
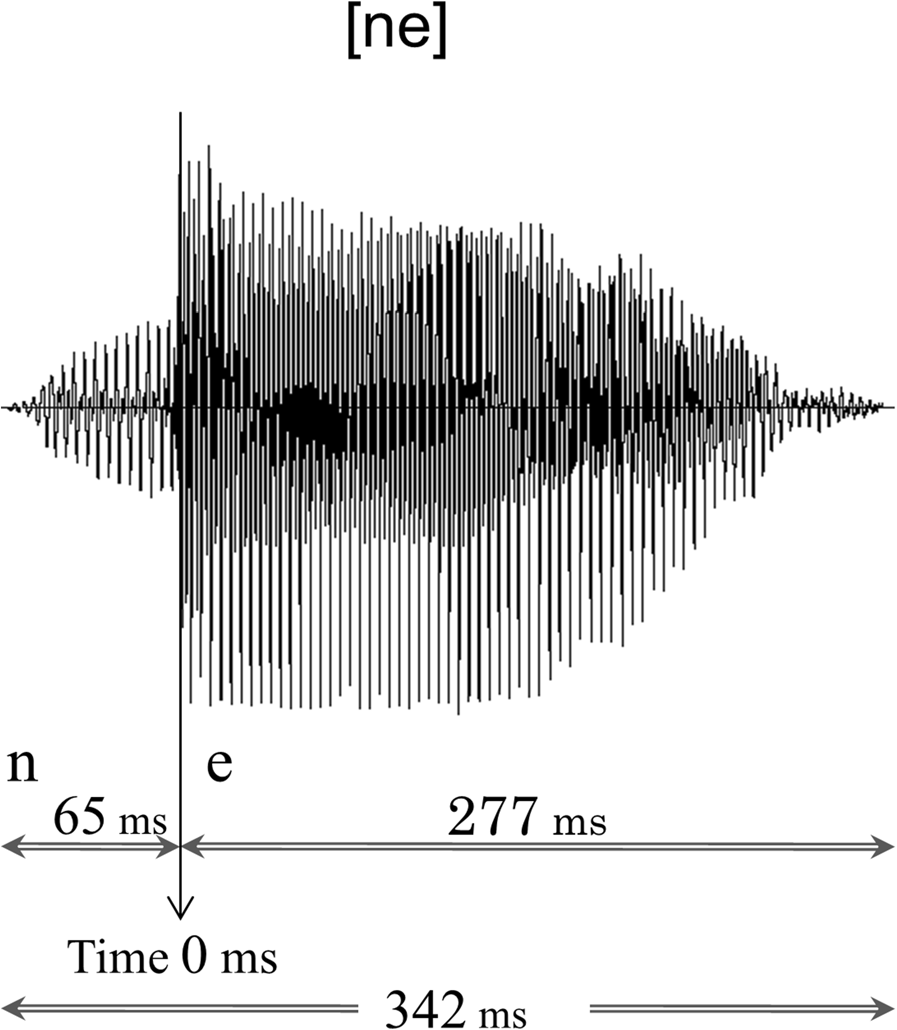
**Waveform of the /ne/ voice stimulus.** The total duration was 342 ms, with 65 ms for the consonant /n/ and 277 ms for the post-consonantal vowel sound /e/. The onset time for the MEG averaging was considered the start of the vowel.

MEG recordings were obtained from all participants during auditory syllable sound stimulation that comprised the Japanese syllable /ne/ [31]. We employed this syllable because /ne/ is one of the Japanese final sentence particles that carry prosodic information [50,51]. The syllable /ne/ is often used in Japanese mother-child conversations and expresses the speaker’s request for acknowledgement or empathy from the listener [52,53]. In the present study, we used typical oddball sequences consisting of standard stimuli at a rate of 83% (456 times) and deviant stimuli at a rate of 17% (90 times). In the standard stimulus, /ne/ was pronounced with a steady pitch contour, whereas in the deviant condition, /ne/ was presented with a falling pitch. Eventually, we adopted only standard stimuli for subsequent ECD estimations because a sufficient number of periods to calculate ECD remained after the rejection of artefacts in all the children. A female native Japanese speaker produced the /ne/ sounds, which were presented using a condenser microphone (NT1-A; Rode, Silverwater, NSW, Australia) on a personal computer. As shown in Figure 2, the duration of the stimulus was 342 ms, and the duration of the consonant /n/ was 65 ms. In this study, the beginning of the vowel sound /e/ was defined as the onset time. The ISI was 818 ms. Both stimuli had a level of approximately 65 dB (A-weighted) against a background noise of 43 dB, which was measured with an integrating sound level meter (LY20; Yokogawa, Tokyo, Japan). The stimulus was presented to participants binaurally through a hole in the MEG chamber using speakers (HK195 Speakers; Harman Kardon, Stamford, CT) that were placed outside of the shielded room. The duration of the recording was 12 min.

### AEF acquisition and analysis

**Figure 3:**
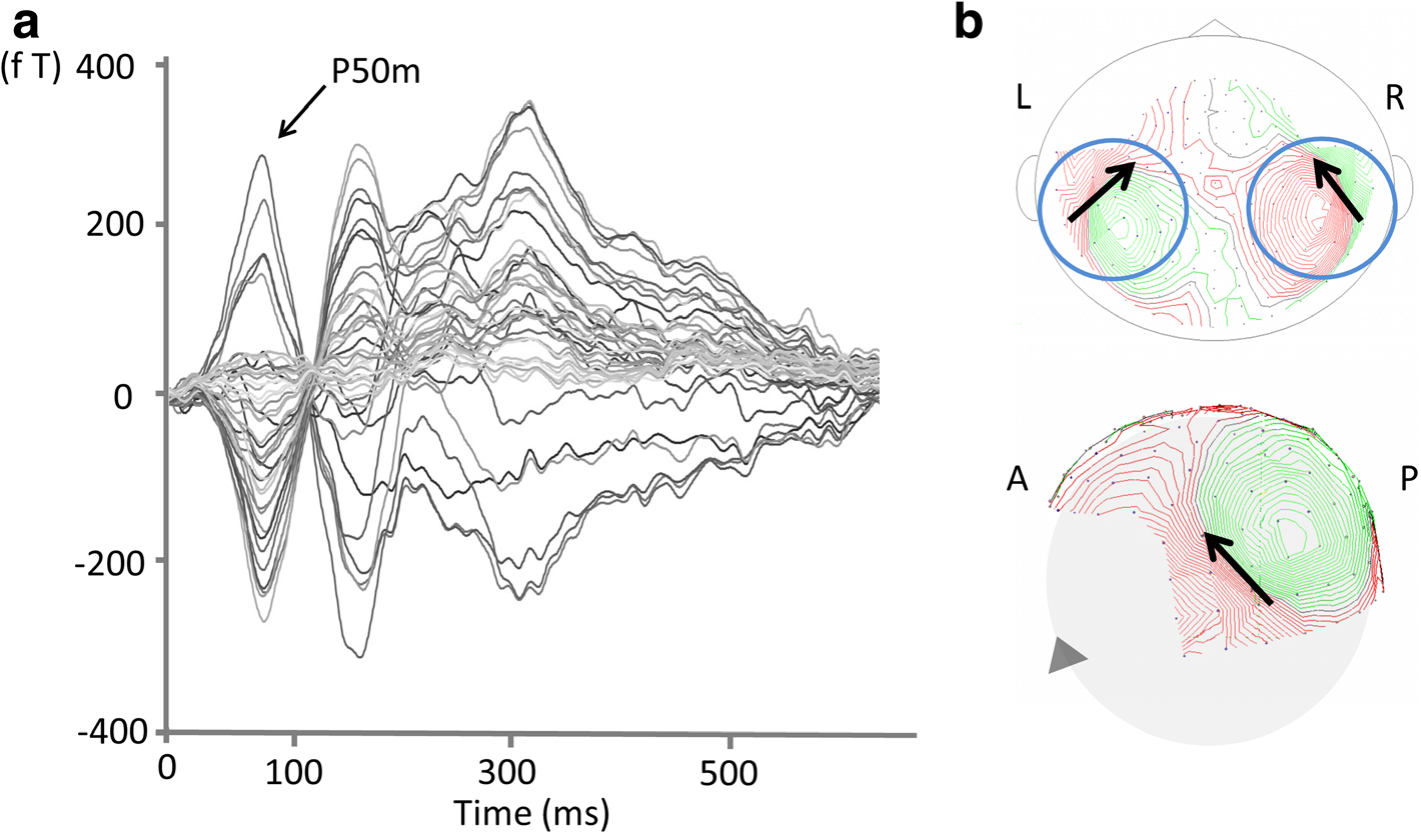
**Source modelling of neuromagnetic responses evoked through auditory stimuli. (a)** The response showed an activity peak at 45–150 ms. **(b)** Typical magnetic field patterns of the peak in the auditory cortex. The magnetic field strength is indicated by a *coloured line*, which varies from *green* (flux-in) to *red* (flux-out). Sensor locations are indicated by *small dots*. *Black arrows* indicate the directions of equivalent current dipole orientation. *Blue open circles* indicate the examples of the 30 sensors for each hemisphere (*left* and *right*) to estimate the current sources. *A*, anterior; *P*, posterior; *L*, left; *R*, right.

The participant’s head was placed in a whole-head Dewar that contained 151 concentrically arranged magnetic sensors. The MEG data were acquired with a sampling rate of 1,000 Hz and were filtered using a 200-Hz low-pass filter. The time series obtained started from the onset of the syllable stimulus at −150 ms and continued to 1,000 ms, and subsequent segments (at least 300 for standard stimuli) were averaged for each of the sensors after baseline correction (−50 to 0 ms) (Figure 3a, b). The segments that were contaminated with artefacts (eye-blink and eye and body movements, typically more than ± 4 pT) were excluded from the analysis. A single ECD model was used to estimate the current sources in the activated cerebral cortex using more than 30 sensors for each hemisphere (left and right) [54]. To estimate the localisation of the current sources, MegLaboratory 160 (Yokogawa/KIT, Kanazawa, Japan) was used.

To identify the P50m component, we accepted estimated ECDs when (1) the goodness of fit (GOF) exceeded 80%; (2) the location of the estimated dipoles using a single ECD model was stabilised within ±5 mm of each coordinate for at least 6 ms during the target response period; (3) the dipole intensities were ≤80 nAm and (4) the direction of the estimated ECD was in an anterosuperior orientation. The latency time point was defined as the maximum estimated dipole intensity value obtained in accordance with the above criteria within a time window of 40 to 150 ms. Regarding the coordinate axes of the P50m component, the centre of a sphere in a spherical model of the volume conductor for the ECD estimation was defined as the origin, and the x-, y- and z-axes represent the leftward direction, the occipital direction and the vertex, respectively.

### Data analysis

Using the Kolmogorov-Smirnov test, it was confirmed that each physiological variable (i.e., raw value of latency and log-transformed intensity) and the scores on the language subtests were normally distributed (*p* > 0.05). For each physiological variable (the log-transformed intensity and latency), a two-way ANOVA was performed (subject group × hemisphere). The between-subject variable was the group (TD vs. ASD), and the within-subject variable was the hemisphere (left and right). Statistical significance was defined as *p* < 0.05. The estimated dipole position of the P50m was also compared for each axis (i.e., the x, y and z directions) between the two groups using a two-way ANOVA (subject group × hemisphere). The between-subject variable was the group (TD vs. ASD), and the within-subject variable was the hemisphere (left and right). For the X direction (the positive and negative values in the row data corresponding to the left and right directions, respectively), the absolute value was used for this comparison. The alpha level was adjusted to 0.05/3 = 0.017 for the statistical analysis because of the multiple comparisons in three axes (i.e., x, y and z).

A hierarchical regression analysis was used to investigate the association between the P50m component (i.e., the log-transformed intensity and latency) and the language-related subscore (i.e., the performance of ‘riddles’). Analyses were performed separately for the right and left hemispheres. Between-person covariates were included in the hierarchical regression analysis as independent factors that were of theoretical importance in a study of brain development and cognitive function. These variables were age (in months) and non-verbal performance (i.e., a combined score of the K-ABC subtests in ‘face recognition’ and ‘hand movement’).

To investigate whether there is a significant change in the explained variance of the model that includes the final independent factor (i.e., the performance of ‘riddles’) compared to the model that includes only the confounding variables (age in months and non-verbal performance), we employed a hierarchical regression model in which the language task performance was included in the final step. The demographic variable (age in months) was entered at the first step in this model. The non-verbal performance was entered at the second step, and the language-related subscore was entered at the final step. The alpha level was adjusted to 0.05/2 = 0.025 for the statistical analysis of the P50m component because of the multiple comparisons in the two hemispheres (i.e., left and right).

## Results

### The profile of the K-ABC subscores in children with ASD and TD children

When we compared the performances on the K-ABC subtests that were common to all ages between the TD children (*n* = 35) and the children with ASD (*n* = 35) (Additional file 1: Figure S1), an unpaired t-test revealed that “riddles” (the verbal reasoning task) was the weakest subtest for the children with ASD (*t* = 2.285; *p* = 0.025). In terms of the scores of the “riddles” (the verbal reasoning task), there were no significant differences between children who achieved a full ADOS score (*n* = 29) and children who did not achieve a full ADOS score (*n* = 6) (*t* = −0.800 *p* = 0.429).

### Detection of the P50m component

One of the 35 children with ASD did not complete the experiment because of boredom and unwillingness to undergo the measurements. Therefore, the MEG data were acquired from 35 TD children and 34 children with ASD. The P50m component could not be detected in one or both hemispheres in two TD children and four children with ASD (i.e., did not meet the criteria for ECD). In total, the bilateral ECD sources were reliably estimated from 33 TD children and 30 children with ASD. Demographic characteristics are shown in Table 1 for the participants whose P50m dipole sources were reliably estimated in both hemispheres.

### P50m intensity

**Figure 4:**
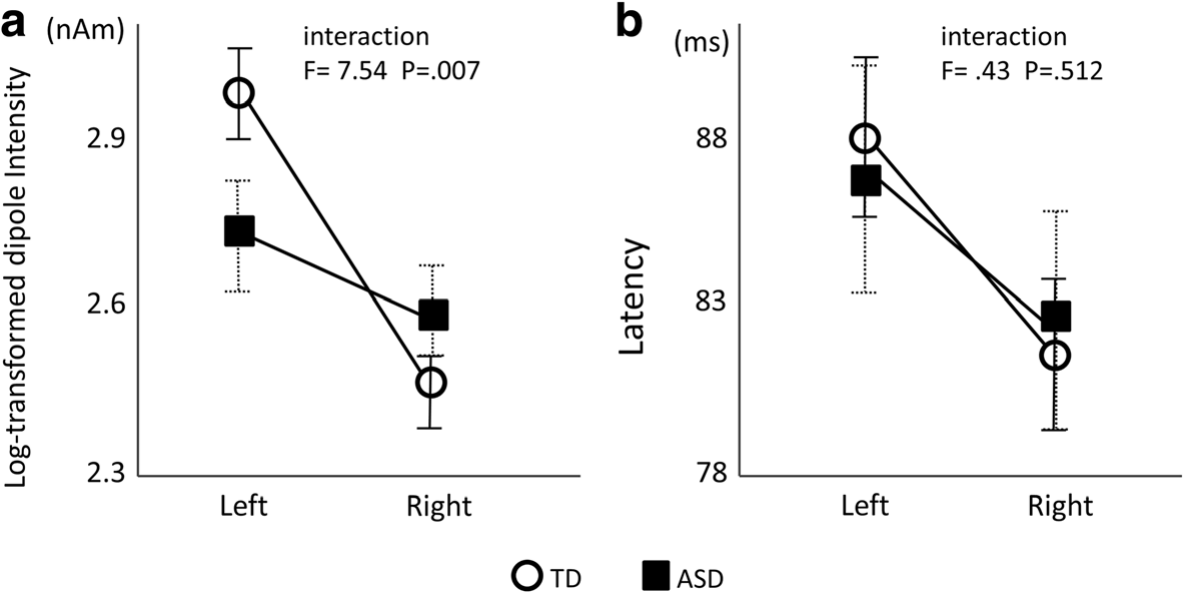
**Intensity (a) and latency (b) of the P50m component.** The *open circles* indicate typically developing children, and the *closed squares* indicate children with ASD. Note the significant interaction between the group and hemisphere (F = 7.54; *p* = 0.007) for the intensity values **(a)** but not for the latency values **(b)**. The values indicate the mean ± SE.

For the TD children, the dipole intensity was 21.6 ± 8.8 and 13.7 ± 6.9 nAm (mean ± SD) in the left and right hemispheres, respectively. For the children with ASD, the dipole intensity was 17.2 ± 7.8 and 14.4 ± 5.6 (mean ± SD) in the left and right hemispheres, respectively. A comparison of the log-transformed intensity using two-way ANOVA (subject group × hemisphere) revealed a significant main effect of the hemisphere (F = 22.36; *p* < 0.0001); however, there were no significant effects of the group. Significant interactions between the group and hemisphere were observed (F = 7.54; *p* = 0.007, Figure 4a). This significant interaction indicates that the children with ASD exhibited significantly less leftward lateralisation compared with the TD children.

As a complementary analysis, we re-calculated the two-way ANOVA (subject group × hemisphere) in only the right-handed participants (TD = 33, ASD = 24) to exclude the confounding effect of handedness. Almost the same results were obtained: there was a significant main effect of the hemisphere (F = 17.52; *p* = 0.0001), there were no significant effects of the group, and significant interactions between the group and the hemisphere were observed (F = 7.54; *p* = 0.0008).

In addition, as a complementary analysis, we re-calculated the two-way ANOVA (subject group × hemisphere) without the six children with ASD who did not meet the criteria of ASD by ADOS (TD = 33, ASD = 25). Almost the same results were obtained: a significant main effect of the hemisphere (F = 19.67; *p* < 0.0001) was observed. There were no significant effects of the group (F = 0.56; *p* = 0.456). Significant interactions between the group and hemisphere were observed (F = 5.88; *p* = 0.018).

### P50m Latency

For the TD children, the mean dipole latency was 88 ms (1 SD = 13) and 81 ms (1 SD = 13) in the left and right hemispheres, respectively. For the children with ASD, the mean dipole latency was 87 ms (1 SD = 19) and 82 ms (1 SD = 18) in the left and right hemispheres, respectively. A comparison of the latency using two-way ANOVA (subject group × hemisphere) revealed a significant main effect of the hemisphere (F = 9.702; *p* = 0.002); however, no significant main effects of the group were observed. Moreover, no significant interactions between the group and the hemisphere were observed (F = 0.434; *p* = 0.512; Figure 4b).

As a complementary analysis, we re-calculated the two-way ANOVA (subject group × hemisphere) in only the right-handed participants (TD = 33, ASD = 24) to exclude the confounding effect of the handedness. Almost the same results were obtained: there was a significant main effect of the hemisphere (F = 7.835; *p* = 0.008), no significant main effect of the group was observed, and no significant interactions between the group and hemisphere were observed (F = 0.722; *p* = 0.399).

In addition, as a complementary analysis, we re-calculated the two-way ANOVA (subject group × hemisphere) without the six children with ASD who did not meet the criteria of ASD by ADOS (TD = 33, ASD = 25). Almost the same results were obtained: there was a significant main effect of the hemisphere (F = 10.47; *p* = 0.002); however, no significant main effects of the group were observed. Moreover, no significant interactions between the group and the hemisphere were observed (F = 0.09; *p* = 0.764).

### Source localisation of the P50m

**Table 2:** Position of the P50m source

	**Left**			**Right**		
	x	y	z	x	y	z
TD	51.61 (7.6)	3.77 (6.0)	−7.85 (8.6)	−56.80 (8.3)	−7.66 (8.8)	−16.35 (16.3)
ASD	48.58 (9.5)	2.96 (9.1)	−11.48 (9.4)	−53.02 (7.7)	−8.42 (7.8)	−14.81 (9.0)

The centre of the hypothetical spherical head model is the origin. The values are the mean (standard deviation).

The positive values on the *x*-, *y*- and *z*-axes indicate the leftward direction, the occipital direction and vertex, respectively.

The results of the two-way ANOVA (subject group × hemisphere) showed a significant main effect of the hemisphere in the x- (F = 10.626; *p* = 0.0018), y- (F = 63.447; *p* < 0.0001) and z-axes (F = 16.539; *p* = 0.001) (Table 2). However, there was no significant main effect of the group in the x-, y- and z-axes, and no significant interaction was observed between the group and the hemisphere in the x- (F = 0.064; *p* = 0.801), y- (F = 2.653; *p* = 0.987) and z-axes (F = 3.159; *p* = 0.080).

### Correlation between P50m intensity and language-related performance

**Table 3:** Summary of the hierarchical regression analysis for P50m log-transformed intensity

		**Left**				**Right**				**N**
		**β**				**β**				
		**Step 1**	**Step 2**	**Step 3**	***t in step 3***	**Step 1**	**Step 2**	**Step 3**	***t in step 3***	
TD	Age (months)	0.172	0.097	0.108	0.331	0.067	−0.532	−0.592	−2.036	33
	Non-verbal performance		0.095	0.097	0.327		0.763*	0.751*	2.846	
	Riddles			−0.018	−0.075			0.104	0.479	
	R	0.172	0.182	0.182		0.067	0.477	0.483		
	Adjusted R^2^	0.030	0.033	0.033		0.004	0.227	0.233		
	ΔR^2^	0.000	0.000	0.000		0.000	0.176	0.154		
ASD	Age (months)	−0.051	0.013	−0.061	−0.284	−0.288	−0.246	−0.294	−1.390	30
	Non-verbal performance		−0.292	−0.392	−1.692		−0.187	−0.251	−1.096	
	Riddles			0.197	0.761			0.126	0.498	
	R	0.051	0.289	0.322		0.288	0.340	0.352		
	Adjusted R^2^	0.003	0.084	0.104		0.083	0.116	0.124		
	ΔR^2^	0.000	0.016	0.000		0.050	0.050	0.023		

*p < 0.025.

Hierarchical regression analyses examining the relationship between the P50m log-transformed intensity (in the right and left hemispheres) and the performance of the K-ABC language subtest ‘riddles’ were conducted separately for the TD children and ASD children. As shown in Table 3, in both the TD children and ASD children, the hierarchical multiple regression model revealed no significant relationships between the K-ABC ‘riddle’ subtest performance and the P50m dipole intensity at any step. However, in the TD children, the ‘non-verbal performance’ reached statistical significance at steps 2 and 3 (final step 3, β = 0.751, *p* = 0.008) in the right hemisphere (Table 3) (an increase in the P50m intensity was correlated with an increased nonverbal performance in the final step). Age was not a statistically significant factor at any step (Table 3) in either the TD children or the ASD children.

As a complementary analysis, we re-calculated the hierarchical regression analysis except six children with ASD who did not meet the criteria of ASD by ADOS (ASD = 25). Almost the same results were obtained: no independent factors reached significance at any step (Additional file 1: Table S1) in children with ASD.

### Correlation between P50m latency and language-related performance

**Table 4:** Summary of the hierarchical regression analysis for P50m latency

		**Left**				**Right**				**N**
		**β**				**β**				
		**Step 1**	**Step 2**	**Step 3**	***t in step 3***	**Step 1**	**Step 2**	**Step 3**	***t in step3***	
TD	Age (months)	−0.463*	−0.657*	−0.358	−1.380	−0.267	−0.599	−0.268	−0.982	33
	Non-verbal performance		0.248	0.307	1.302		0.423	0.488	1.972	
	Riddles			−0.521*	−2.681			−0.577*	−2.823	
	R	0.463	0.487	0.624		0.267	0.374	0.570		
	Adjusted R^2^	0.214	0.237	0.389		0.071	0.140	0.325		
	ΔR^2^	0.189	0.187	0.326		0.041	0.082	0.255		
ASD	Age (months)	−0.058	−0.100	−0.122	−0.553	−0.435*	−0.503*	−0.583*	−3.092	30
	Non-verbal performance		0.192	0.162	0.677		0.308	0.199	0.974	
	Riddles			0.058	0.218			0.214	0.935	
	R	0.058	0.196	0.201		0.435	0.528	0.550		
	Adjusted R^2^	0.003	0.038	0.040		0.189	0.279	0.302		
	ΔR^2^	0.000	0.000	0.000		0.160	0.226	0.222		

*p < 0.025.

**Figure 5:**
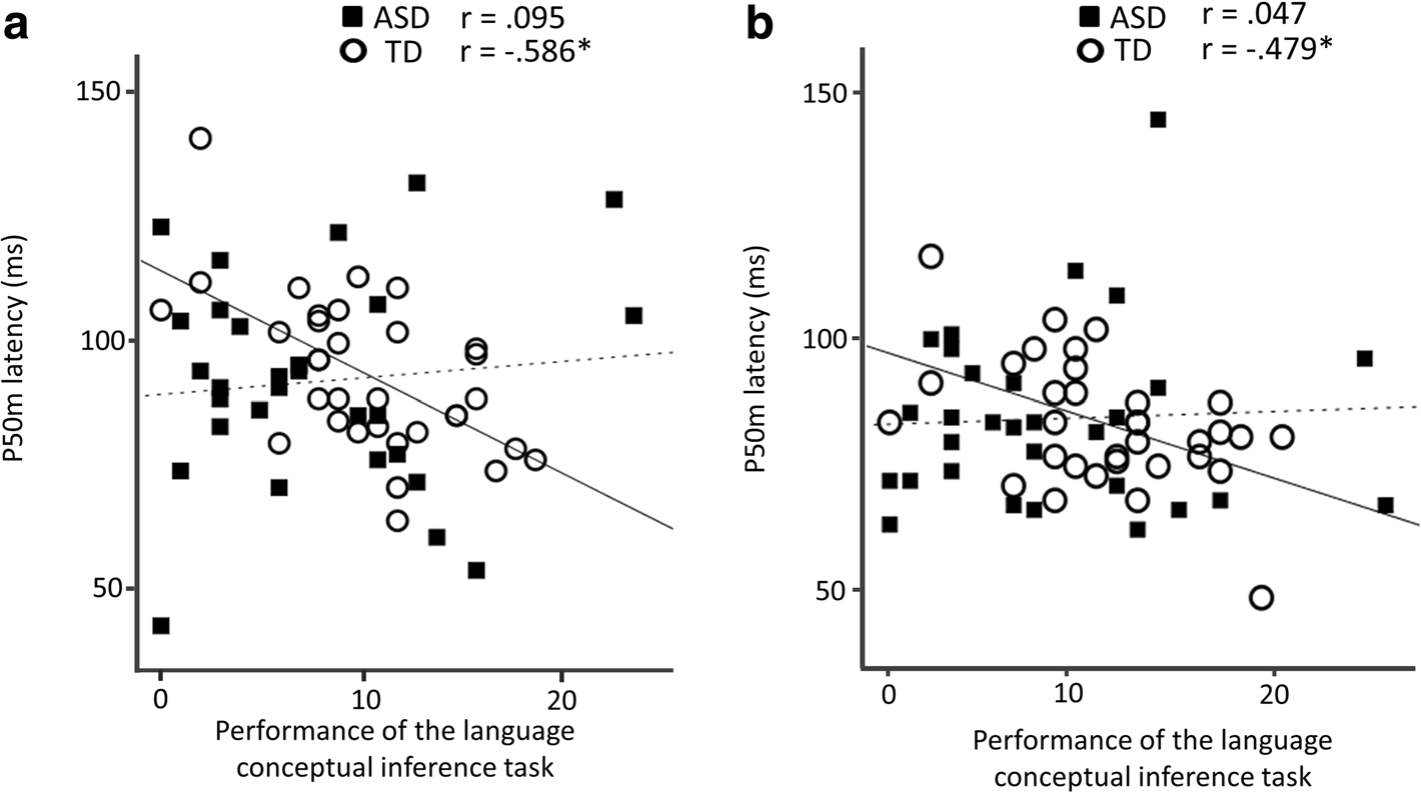
**Scatter plot of the P50m latency and the performance of language conceptual inference in both groups.** For the TD children, a shorter P50m latency in both hemispheres was associated with a higher performance on the language conceptual inference tasks in the left (**a**: *p* = 0.0002) and right (**b**: *p* = 0.0042) hemispheres. There was no significant correlation between the P50m dipole latency and the performance on the language conceptual inference tasks for the children with ASD. *Solid line*, regression line for the TD children; *broken line*, regression line for the children with ASD.

**Figure 6:**
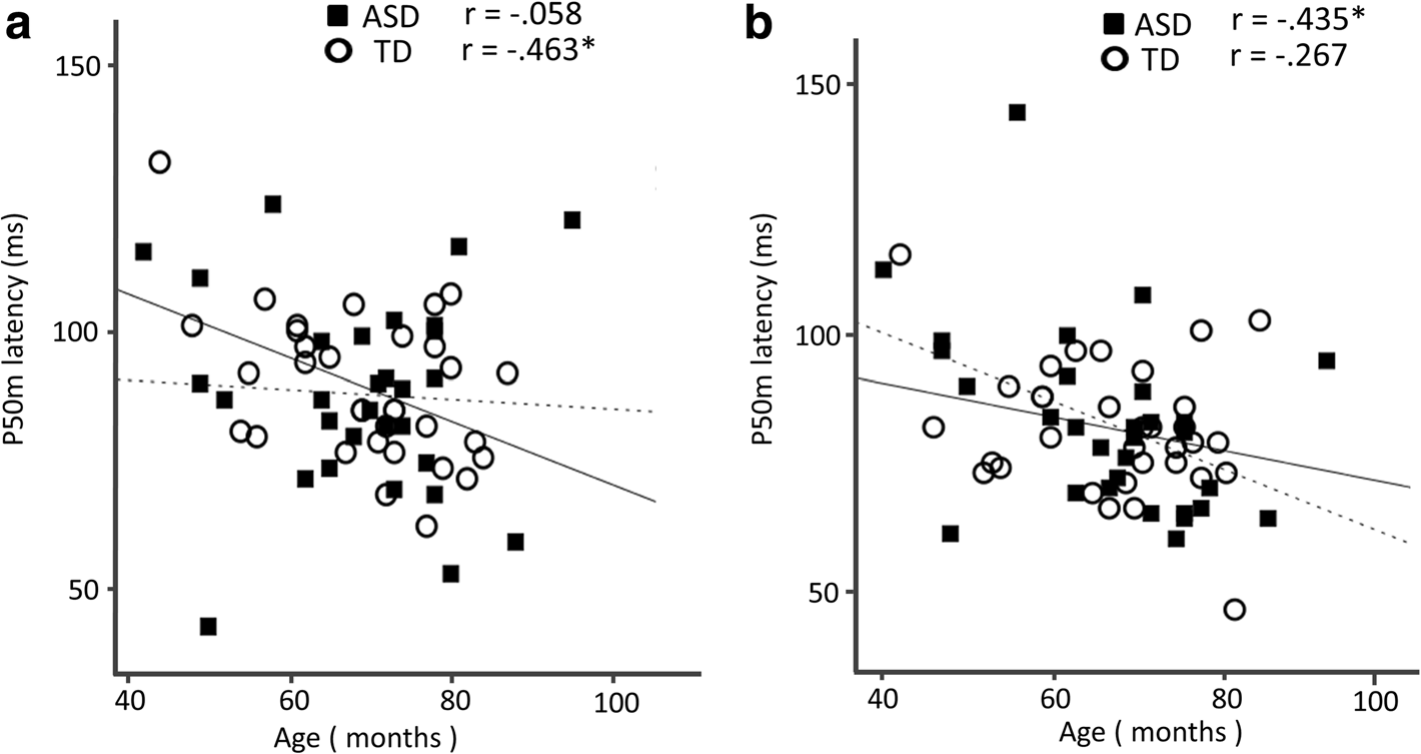
**Scatter plot of the P50m dipole latency and age, in both groups.** For the TD children, a shorter P50m latency in the left hemisphere was associated with a higher age (**a**: *p* = 0.0061). By contrast, for the children with ASD, a shorter P50m latency in the right hemisphere was associated with a higher age (**b**: *p* = 0.0156). *Solid line*, regression line for the TD children; *broken line*, regression line for children with ASD.

A hierarchical regression analysis examining the relationship between the P50m latency (in the right and left hemispheres) and the performance on the K-ABC language subtest ‘riddles’ was conducted separately for the TD children and ASD children. As shown in Table 4, in the TD children, the hierarchical multiple regression model revealed that the K-ABC ‘riddle’ language subtest performance was significantly and independently associated with the P50m latency in both the left (final step 3, ΔR^2^ = 0.326, β= −0.521, *p* = 0.012) and right hemispheres (final step 3, ΔR^2^ = 0.255, β= −0.577, p = 0.009) (a decrease in the P50m latency was correlated with higher verbal performance in the final step), whereas in the children with ASD, no significant relationships between the K-ABC ‘riddle’ subtest performance and the P50m latency were demonstrated at any step.

In addition, for the TD children, there was a significant relationship between the P50m latency in the left hemisphere and the age in months during the first two steps; however, this significance diminished at step 3 (Table 4). In the right hemispheres of the children with ASD, a relationship between P50m latency and age maintained statistical significance until the final step (step 3, β= −0.583, *p* = 0.005) (i.e., a decrease in the P50m latency was correlated with a higher age in months in the final step). The relationship between the P50m latency and the ‘non-verbal performance’ did not reach statistical significance at any step (Table 4) in both the TD children and ASD children.

As a complementary analysis, we re-calculated the hierarchical regression analysis without the six children with ASD who did not meet the criteria of ASD by ADOS (ASD = 25). Although the same tendency was observed, the relationship between P50m latency and age was diminished at all steps (Additional file 1: Table S2), and no independent factor reached significance at any step (Additional file 1: Table S2) in children with ASD.

In addition, as a complementary analysis for the relationships in which the significance was observed at the final step in the first analysis (TD = 33, ASD = 25), Pearson’s correlation coefficients (i.e., simple linear regression) were calculated separately between the P50m latency and the verbal performance (Figure 5) or the age in months (Figure 6) in the left and right hemispheres in the TD children and ASD children. As shown in Figure 5a and b, a significant negative correlation between the P50m dipole latency and the verbal performance was observed in the left (r = −0.586, *p* < 0.001) and the right (*r* = −0.479, *p* = 0.005) hemispheres in the TD children, whereas no significant correlation was observed in the children with ASD in either hemisphere. As shown in Figure 6a and b, a significant negative correlation between P50m dipole latency and age in months was observed in the left hemisphere of the TD children (*r* = −0.463, *p* = 0.007), whereas this negative correlation was observed in the right hemisphere of the children with ASD (*r* = −0.435, *p* = 0.016).

## Discussion

This study is the first report to describe the features of bilateral auditory evoked field (AEF) dipole source features in young children (3–7 years old) with ASD. Using MEG devices customised for children, we replicated our previous study for 2- to 5-year-old TD children (*n* = 59; five participants were the same as in the present study [31]), which demonstrated leftward lateralisation in the P50m dipole intensity during binaural human voice stimulation in TD children. In addition, our present result revealed that the children with ASD showed less leftward lateralisation in the intensity of the P50m during short ISI human voice stimuli compared with TD children. Interestingly, as a result of the multiple regression analysis to investigate the relationship between the P50m component and language performance, a shorter P50m latency in both hemispheres was specifically correlated with higher language-related performance in the TD children. This result suggested that the human voice evoked the P50m component in both hemispheres, as reflected by the brain maturation in structures related to language acquisition. Given that myelination considerably enhances nerve conduction velocity and leads to a shorter latency of the brain response [55-58], a shorter P50m latency to human voice stimuli might reflect the progression of increasing myelination in the brain areas related to human voice processing. In contrast, the P50m latency in children with ASD did not show any correlation with language-related performance.

Functional brain organisation is thought to be shaped in an activity-dependent fashion by a complex interaction between multiple genes and epigenetic factors [59]. The language processing area is one of the most interesting regions in which a correlation between myelination and language ability has been reported in young children [39]. Our results suggest that auditory-related brain organisation in TD children is shaped in a language-ability-dependent fashion that is also affected by chronological age. In children with ASD, however, we failed to demonstrate an ability dependence of the P50m component. This result suggests that, in the right hemisphere, auditory-related brain organisation in ASD is shaped in a language-ability-independent fashion while being affected by chronological age. Although further study is necessary to demonstrate what type of ability is associated with the P50m component in children with ASD, this language-related functional discrepancy may shed light on the neural basis of ASD.

In the present study, increasing chronological age was associated with shorter P50m latency in the right hemisphere in ASD but not in TD children, from a hierarchical regression analysis. As reported in the previous EEG study, with 118 normal subjects, the P1 (i.e., P50m in the MEG study) latency decreases with age between 5 and 20 years of age [24], and it therefore appears to be natural for both TD and ASD children that increasing chronological age is associated with shorter P50m latency. In the present study, no statistical significance of the regression analysis in TD children could be attributed to the narrower range in the age (i.e., 3 to 7 years old), whereas significant results observed in children with ASD may be due to the accelerated maturation of white matter in young children with ASD within such a narrow age range [60].

In contrast to the TD children, the present results revealed that children with ASD showed a less left-lateralised P50m during human voice stimuli (Figure 4a). This atypical brain lateralisation during human voice stimuli is consistent with recent MEG studies in older children with ASD (8–17 years old) [61]. These authors demonstrated rightward lateralisation in the AEF late component during vowel stimuli in relatively small samples (*n* = 6), which suggests that children with ASD and TD children follow opposite maturational trajectories in language-related brain lateralisation. Our results provide further evidence in younger children with ASD during conscious conditions. In addition, our results (less leftward lateralisation in children with ASD) were supported by those obtained from recent brain volumetric studies using MRI, which revealed an aberrant rightward lateralisation [15,16,22,62]. Intriguingly, the rightward asymmetry of the cortical volume in the auditory association area (i.e., posterior superior temporal gyrus and planum temporale) was shown in children with ASD [16,22]. Thus, these results are consistent with our results and showed less leftward lateralisation in the P50m intensity, which is generated in these areas [63].

In the correlation analysis for P50m intensity and language performance, our current study failed to demonstrate a significant correlation, which we had previously found in 2- to 5-year-old children [31]. The discrepancy between these studies might reflect the older and wider age range of the subjects (3 to 7 years old) in the present study. Previous EEG studies demonstrated that the growth curve of the P50 amplitude (but not the latency) in young children followed an inverted U-shaped curve. These authors reported that the amplitude of the P50 was the largest in toddlers (1 to 2 years old) compared with newborns (< 7 days) and young children (4 to 6 years old) [27] and that it decreased with age from childhood to adulthood [24,64]. Judging from these results, linear regression models are not always suitable for comparing the P50m intensity and age in young children. Incidentally, in the present study, an unexpected association between the P50m dipole intensity in the right hemisphere and non-verbal performance was also demonstrated in TD children. However, caution must be exercised in drawing any definitive conclusions from this multiple regression model using the P50m intensity and the age in months because of the same reasons mentioned above (i.e., linear regression models might not be suitable).

Although no previous MEG study has focused on the P50m in preschool (< 7 years old) children with ASD, there are a few P50m studies in school-age children and adolescents with ASD [25,42,65]. One previous study investigated the relationship between clinical language impairment and the latency of auditory P50m and N100m in 7- to 18-year-old subjects with various clinical conditions (i.e., typical development, ASD or specific language impairment) and demonstrated that longer P50m latency in the right hemisphere was the best predictor of clinical language impairment [65]. However, we observed similar relationships (i.e., longer P50m latency correlated with lower language performance) in both hemispheres in the TD children but not in the children with ASD. The other study investigated the P50m and N100m only in the left hemisphere in school-age children and adolescents with ASD and failed to demonstrate significant differences in either the latency or amplitude between TD and ASD children [25], which was not inconsistent with our results in the left hemisphere.

In this study, we showed that the locations of the ECDs for P50m were more anterior in the right hemisphere than in the left hemisphere in both groups (Table 2). This trend is consistent with previous studies that have also demonstrated the asymmetry of P50m locations in 6- to 9-year-old TD children [30,66]. With regard to P50m, the present study is the first to show the P50m source position in young children with ASD and to demonstrate that both TD and ASD children similarly displayed right-sided P50m anteriority. With regard to N100m, a previous study demonstrated right-sided N100m anteriority in 8- to 15-year-old TD children, whereas children with ASD did not show asymmetry [43]. However, we did not examine the N100m component in this study because of a lower GOF in the ECD estimation in 2- to 5-year-old children [31].

The present study had some general limitations. First, we investigated the P50m using only one type of auditory stimulus (the human voice /ne/). Therefore, we cannot determine whether our results are specific to human voice stimuli. Second, we analysed the brain’s response to auditory stimuli only. Therefore, we cannot generalise this reduction in leftward brain lateralisation in children with ASD to other sensory modalities [19] or multisensory stimuli [67]. Third, a majority of the children with ASD in the present study were high-functioning subjects and therefore may not represent children with ASD who have a comorbid language disability. Fourth, the participants were monitored using a video camera, which facilitated detection of obvious body movement. The examiner who accompanied the children in the shielded room also instructed the participants to maintain the position of their head throughout the experiment. However, the influence of fine head movements might have been a confounding factor in the present study. Fifth, we employed a cross-sectional rather than a longitudinal design. Future research with a longitudinal design is crucially necessary to provide a better understanding of functional maturations in the auditory cortex in children with ASD. In spite of these limitations, the present study is the first to demonstrate atypical brain maturation related to P50m generation in young children with ASD.

## Conclusions

Child-customised MEG devices facilitated the acquisition of whole-brain functional measurements during conscious conditions in young children. Children with ASD (most of whom were high-functioning subjects) showed less leftward lateralisation of the intensity of the P50m component in response to voice stimuli compared with TD children. Regardless of their language development, our results suggest that there is atypical brain function in the auditory cortex of young children with ASD.

## Abbreviations

AEF: Auditory evoked magnetic field; ASD: Autism spectrum disorder; TD: Typical developing; ECD: Equivalent current dipole; EEG: Electroencephalography; ISI: Interstimulus interval; K-ABC: Kaufman Assessment Battery for Children; MEG: Magnetoencephalography; SLI: Specific language impairment.

## Competing interests

The authors declare no competing interests.

## Authors’ contributions

YY participated in the conception and design of the study, performed the statistical analysis and wrote the first draft of the manuscript. MK participated in the conception design, and coordination of the study and wrote the final draft of the manuscript. KS and SU participated in the conception of the study and operated the child-customised MEG system. TM, YN, TTa and MS recruited participants in this study from their facilities. YO, TTs, MO and GBR participated in the conception of the study and advised YY regarding the assessment of the ASD and analysis. YH advised KS and SU regarding the operation of the child-customised MEG system. HH and YM participated in the conception of this study and took chief responsibility for the study. All the authors read and approved the final manuscript.

## Supplementary Material

Additional file 1 Figure S1 Summary of the hierarchical regression analysis for the log-transformed intensity of P50m. The performance of children with ASD and young TD children on each K-ABC subtest (mean score ± SD) is shown for tests applicable to all ages. An unpaired *t*-test revealed significantly lower performance in children with ASD compared with TD children on the riddle task, which tests “language conceptual inference ability”. *P<0.05. **Table S1.** Summary of the hierarchical regression analysis for the log-transformed intensity of P50m. *p < .025. **Table S2.** Summary of the hierarchical regression analysis for P50m latency. *p < .025. Click here for file
