# Supplementary material for: Atypical brain lateralisation in the auditory cortex and language performance in 3- to 7-year-old children with high-functioning autism spectrum disorder: a child-customised magnetoencephalography (MEG) study
Source: Mol Autism. 2013 Oct 8;4:38. doi: 10.1186/2040-2392-4-38 (PMC4021603; doi:10.1186/2040-2392-4-38)
Supplement: Additional file 1 Figure S1 — Summary of the hierarchical regression analysis for the log-transformed intensity of P50m. The performance of children with ASD and young TD children on each K-ABC subtest (mean score ± SD) is shown for tests applicable to all ages. An unpaired t-test revealed significantly lower performance in children with ASD compared with TD children on the riddle task, which tests “language conceptual inference ability”. *P<0.05. Table S1. Summary of the hierarchical regression analysis for the log-transformed intensity of P50m. *p < .025. Table S2. Summary of the hierarchical regression analysis for P50m latency. *p < .025. [file 2040-2392-4-38-S1.doc]

Molecular Autism

Supplemental information

Title: Atypical brain lateralisation in the auditory cortex and language performance in 3- to 7-year-old children with autism spectrum disorder: a child-customised magnetoencephalography (MEG) study

**
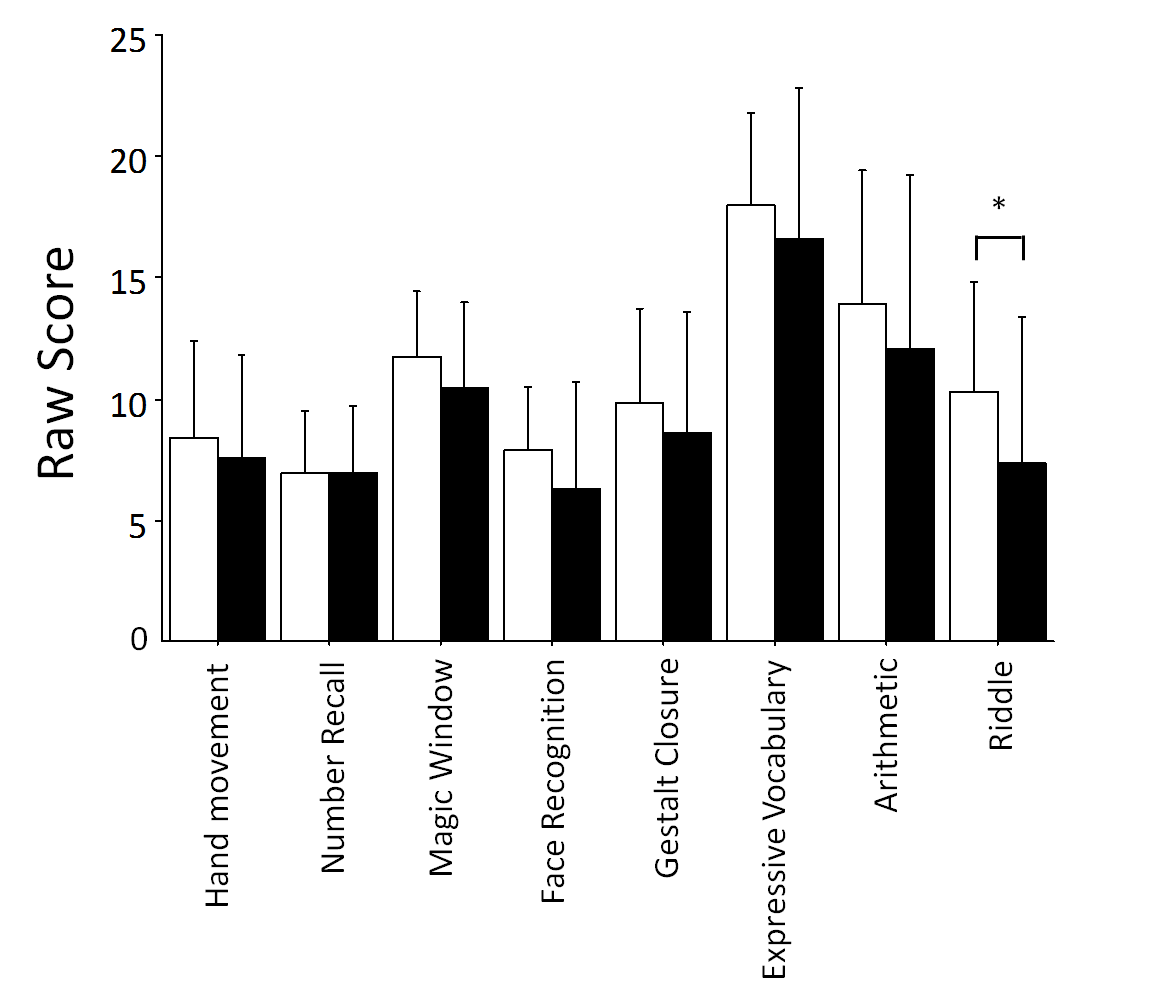
**

**Figure S1**

Figure S1. The performance of children with ASD and young TD children on each K-ABC subtest (mean score ± SD) is shown for tests applicable to all ages. An unpaired *t*-test revealed significantly lower performance in children with ASD compared with TD children on the riddle task, which tests “language conceptual inference ability”. **P*<0.05.

**Tables S1-S2**

Table S1. Summary of the hierarchical regression analysis for the log-transformed intensity of P50m.

*p < .025

Table S2. Summary of the hierarchical regression analysis for P50m latency.

* p < .025
